# In vitro antibacterial effect of carbamide peroxide on oral biofilm

**DOI:** 10.3402/jom.v5i0.20392

**Published:** 2013-06-12

**Authors:** Chao Shu Yao, John Douglas Waterfield, Ya Shen, Markus Haapasalo, Michael I. MacEntee

**Affiliations:** University of British Columbia, Vancouver, BC, Canada

**Keywords:** antibacterial, biofilm, carbamide peroxide, confocal laser scanning microscopy

## Abstract

This study compared the effects of carbamide peroxide (CP) and chlorhexidine (CHX) on oral biofilm in vitro. Collagen-coated hydroxyapatite discs were inoculated with subgingival plaque. After 3 weeks, the emergent biofilms were subjected to 1-, 3-, and 10-min exposures of a 1% CHX gel, a 5% CP gel and rinse, and a 10% CP gel and rinse. Subsequently, the biofilms were stained using a two-colour fluorescent dye kit for confocal laser scanning microscopy, and the volume ratio of dead bacteria to all bacteria was analysed. Compared to a non-treated gel control, the active agents killed bacteria on all the discs, with higher concentration and longer exposure times killing more bacteria. The rinse form disrupted the biofilm quicker than the gel form. Overall, 10% CP showed more disruption of biofilm and a greater proportion of killed bacteria than 1% CHX (*p*<0.05).

Frail elders with difficulties performing oral care are burdened with an increased oral microbial load, which increases their health risks. Gram-negative bacteria are frequently found in the mouths of frail elders, and in the blood of patients with bacteraemia (1, 2). The pathogens isolated from patients with bacteraemia and pneumonia have been genetically matched to those in their oral biofilms (3, 4).

Chlorhexidine (CHX) is commonly used as a mouth rinse to reduce gingival inflammation by damaging bacterial cell walls, precipitating cell contents, and inhibiting microbial accumulation on dental surfaces (5). However, it has only a modest effect in reducing the relative risk of respiratory tract infection (6) and there is insufficient evidence to support its prophylactic use against infective endocarditis (7).

Hydrogen peroxide (HP) was first reported for intraoral use in 1913 to decrease plaque formation (8), and it has been the treatment of choice for acute necrotising ulcerative gingivitis (9) and pericoronitis (10). HP is a common metabolite produced by mammalian and bacterial cells as a microbicidal and a regulatory agent. It is a mild oxidising agent that is reduced via Fenton reaction to a hydroxyl radical (^•^OH) at micromolar concentrations in the presence of intracellular transition metals, such as ferrous ions (Fe^2+^). This reaction can cause cellular damage through the degradation of DNA and oxidation of proteins and lipids (11). At low (<3 mM) concentrations, HP damages DNA and proteins, while at higher (>30 mM) concentrations, it damages bacterial membranes without inducing mutation (12). HP also triggers the release of extracellular DNA without autolysis, which promotes intra-species cell-to-cell adherence and aggregation within the structure of the biofilm (13).

In saliva, HP constitutes the peroxidase–thiocyanate–hydrogen peroxide system, which damages bacterial cell membranes, inhibits glycolysis of *Streptococcus mutans*, and also inhibits further production of HP by streptococci (14). Exogenous HP in saliva also generates hypothiocyanate and exponentially reduces acid production in the biofilm (15). Several streptococci, including pneumococci, produce HP that inhibits the growth of both the host bacteria and other bacteria (16).

HP exhibits a differential effect on human macrophage phagocytosis, with increased activity at low concentrations and suppressed activity at high concentrations (17). Different bacteria exhibit different levels of susceptibility towards HP. While some periopathogens, such as *Aggregatibacter actinomycetemcomitans*, are resistant to HP, pathogens associated with infective endocarditis and pneumonia are susceptible to submicromolar concentrations of HP (16, 18).

HP is not stable at room temperature. Carbamide (urea) peroxide (CP) decomposes into one part HP and two parts urea. Urea further decomposes into ammonia and carbon dioxide via urease activity. HP and CP are acidic, at pH's 4.5 and 5.3, respectively, and exert inhibitory effects on acidophilic pathogens, such as *S. mutans*, through the release of ammonia and elevation of the pH when CP decomposes (19). The neutralising effects of 10% CP can last approximately 2 hours (20), which suggests that it might disturb biofilm more effectively than HP.

Some bacteria produce HP as part of a self-regulatory mechanism and by interspecies competition (16). Therefore, it is unlikely that these bacteria will develop resistance against it, which is an advantage for immunocompromised patients. It causes little ill-effects in the mouth unless there is a hereditary deficiency of catalase or glucose-6-phosphate (21). HP up to 3% and CP up to 15% have been accepted as oral antiseptic agents by the US Food and Drug Administration (22).

There is a renewed interest in HP as an antimicrobial agent against periodontal disease (23), although its ability to reduce the formation of oral biofilm is unclear (24). Most microbiological studies have been conducted on submolar concentrations of HP, and the effects of bacterial enzymes, including catalase, on high levels of HP are unknown (25). Even more limited studies were carried out on the antibacterial effect of CP. A 10% CP gel applied to the teeth of institutionalised patients reduced plaque deposits with no adverse effects (26). An in vitro experiment found that 10% CP gel inhibits the growth of lactobacilli and *S. mutans*, while in vivo, the gel reduced the number of lactobacilli in saliva with no noticeable effect on the *S. mutans* (27).

We hypothesise that CP gels and rinses at 5% and 10% concentrations, respectively, have comparable or superior bactericidal and dislodging effects on the oral biofilm to a control and a 1% CHX gel. The objectives of this study, therefore, were to compare the antibacterial effects of CHX and CP, on oral biofilm cultured in vitro under anaerobic conditions, at concentrations of CP above the level produced by phagocytes but at a level approved for intraoral use.

## Materials and methods

### Antimicrobial agents

CP was prepared from 97% stock powder (Sigma-Aldrich, St. Louise, MO, USA), and CHX from 20% stock solution (Sigma Chemical Co.), by diluting with distilled water to the desired concentrations. Gels were prepared by mixing the solution with 83% amylopectin and 17% amylose to the required concentrations for each agent. The negative control (CG) was made from amylopectin–amylose mixture alone.

### Production of biofilm cultures

The culture for biofilm followed the protocol described by Shen et al. (28). Sterile hydroxyapatite (HA) discs (Clarkson Chromatography Products, Williamsport, PA, USA), 9.7 mm in diameter, 1.5 mm in thickness, were coated with bovine dermal type I collagen – 10 g/mL collagen in 0.012N hydrochloric acid in water (Cohesion, Palo Alto, CA, USA) – by overnight incubation at 4°C in the wells of a 24-well tissue culture plate containing 2 mL of the collagen solution. Supragingival human plaque was suspended in brain heart infusion (BHI) broth (Becton Dickinson, Sparks, MD, USA), to achieve a minimum bacterial cell concentration of 3.2 × 10^7^ CFU/mL. The coated HA discs were rinsed and immersed in the wells of a 24-well tissue culture plate containing 2 mL of the BHI–plaque suspension and incubated anaerobically (AnaeroGen; Oxoid, Hampshire, UK) at 37°C. The media were renewed each week.

### Antimicrobial treatment and determination of efficacy

After 3 weeks, the discs were rinsed with phosphate-buffered saline for 1 min. Photographs of each disc before treatment were taken with a digital camera (Nikon Coolpix 995; Nikon Canada, Mississauga, ON, Canada) mounted on a light microscope (Leica MZ 6; Leica Microsystems Inc., Concord, ON, Canada) under original magnification of 6.3×. Subsequently, the discs were immersed in 2 mL of freshly prepared CG or agents consisting of 5% and 10% CP gel or rinse, or 1% CHX gel for 1, 3 or 10 min.

#### Determination of biofilm removal

After applying the control or the agents, each disc was rinsed with phosphate-buffered saline for 3 min. The LIVE/DEAD BacLight Bacterial Viability Kit L-7012 (Molecular Probes, Eugene, OR, USA) for confocal laser scanning microscopy (CLSM) contains separate vials of the component dyes (SYTO 9 and propidium iodide). The dyes were mixed in a 1:1 solution and applied to the biofilm discs according to the manufacturer's instructions. Post-treatment photographs of the discs were taken at the same camera and microscope settings. The area of biofilm remaining on each disc was mapped and calculated using ImageJ software (Research Services Branch, National Institute of Mental Health, Bethesda, MD, USA) and the average measurement calculated from five discs.

#### Determination of bactericidal efficacy

Five random areas on each disc were scanned and fluorescence from the stained cells was viewed with a CLSM using a 10× lens (Nikon Eclipse C1; Nikon Canada). The excitation/emission maxima for LIVE/DEAD BacLight dyes are 480/500 nm for the SYTO 9 stain and 490/635 nm for propidium iodide. Simultaneous dual-channel imaging displayed green-and-red fluorescence, and images of the scanned biofilm were acquired by the software EZ-C1 v. 3.40 build 691 (Nikon Canada) at a resolution of 512 × 512 pixels. Individual biofilm image covered an area of 1.64 mm^2^ per field-of-view. The confocal scanning was set to 0.5 µm/stack and between 50 µm and 75 µm of the biofilm was scanned for each sample area. Confocal LIVE/DEAD images were analysed by using the colour segmentation algorithm in the bioImage package (www.bioimagel.com©) to separate signals from the red (dead cells) and green (viable cells) fluorescence by colour threshold, and measure the total volume (pixels^3^) covered by each colour. The volume ratio of red to red-and-green fluorescence indicated the proportion of cells killed by each agent. The average of 15 scanned areas from three discs was determined to estimate the bactericidal effects of the control and each agent.

The pH of CG and test agents (CHX and CP) before and after each test period was measured with a Symphony pH Meter SB70P (VMR International, Radnor, Philadelphia, PA, USA) to assess the changes upon interaction with biofilm.

### Statistical analysis

Comparisons between groups were analysed with student's *t*-test at a significance level of *p*<0.05.

## Results

### Biofilm removal

**Fig. 1:**
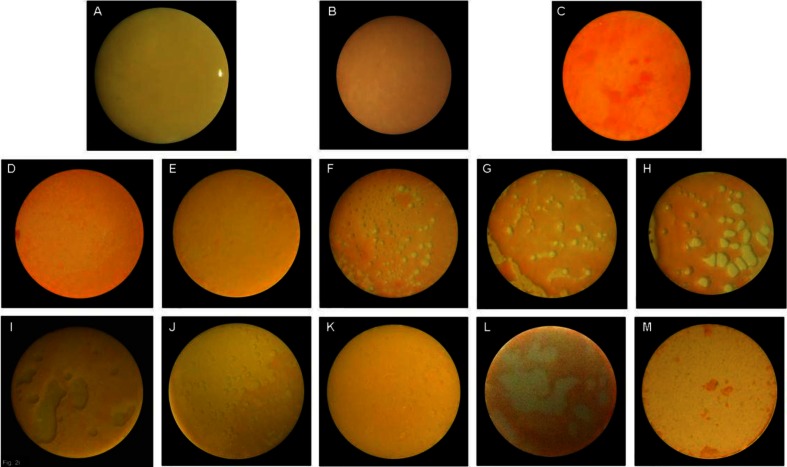
(A) Biofilm on HA disc after 3 weeks of incubation under anaerobic conditions. (B) After exposure to control gel and stained with LIVE/DEAD BacLight Bacterial Viability Kit L-7012. Stained biofilm after exposure to 1% CHX gel for (C) 1 min, (D) 3 min, and (E) 10 min. Stained biofilm after exposure to 5% CP gel for (F) 1 min, (G) 3 min, and (H) 10 min. Stained biofilm after exposure to 10% CP gel for (I) 1 min, (J) 3 min, and (K) 10 min. Stained biofilm after exposure to CP rinse for 1 min at (L) 5% and (M) 10%.

**Fig. 2:**
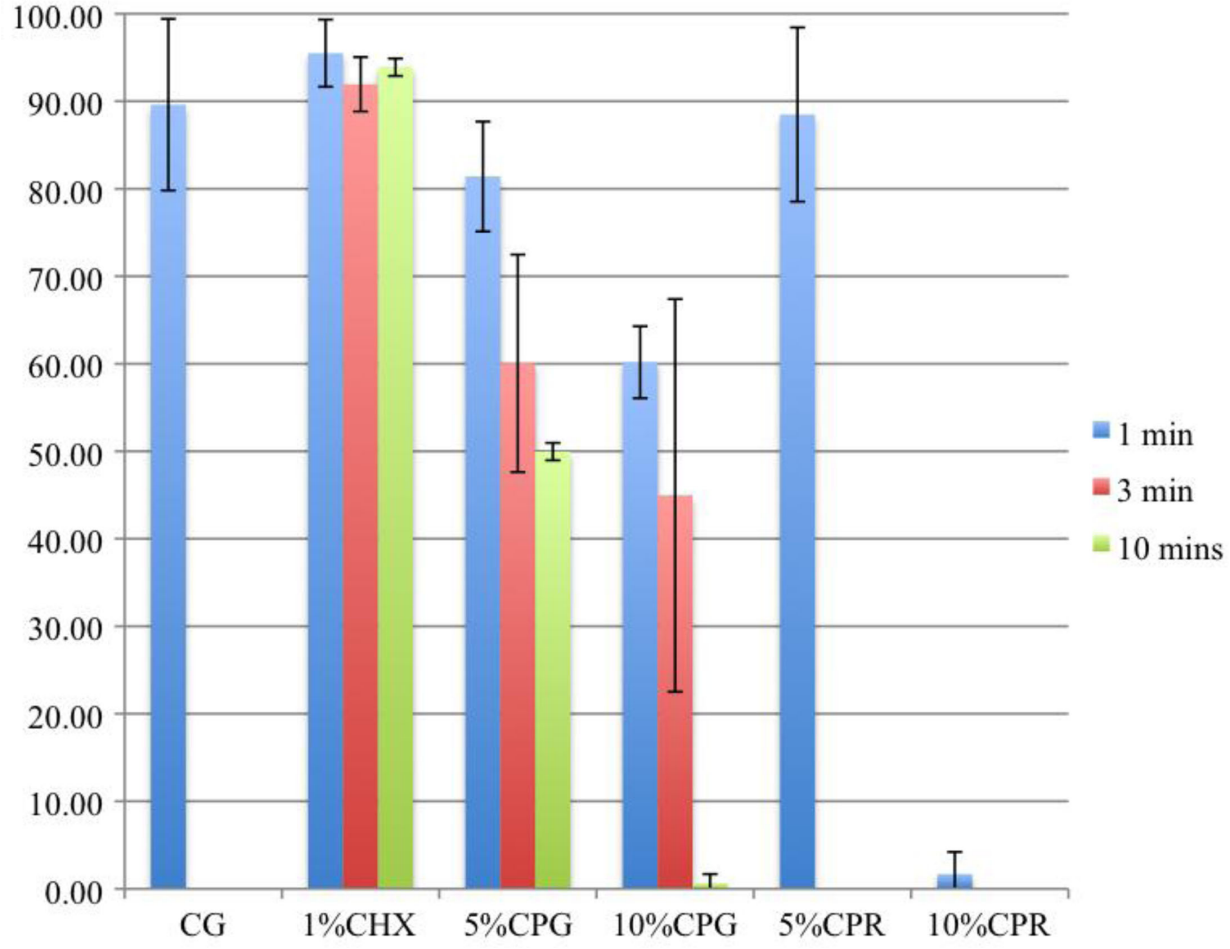
Bar graph representing the percentage of remaining biofilm.

The HA discs were covered almost completely by a biofilm layer after 3 weeks of culturing with supragingival plaque–BHI suspension under anaerobic conditions, and undisrupted by the CG (Fig. 1A and B). The CP gel and rinse, unlike the CHX gel, produced effervescence, which appeared to play a role in detaching the biofilm from the HA discs (Fig. 1C–M). The concentration, exposure time, and form of the CP agents influenced the detachment of biofilm (Table 1 and Fig. 2). Detachment by the 10% gel increased with time; but the effectiveness of the 5% CP gel seemed to diminish after 3 min. Comparing between gel and rinse, the 5% CP gel was more effective than the 5% CP rinse; however, the 10% CP gel required as long as 10 min to achieve the same amount of detachment of the biofilm as the 10% CP rinse after 1 min. The 10% CP gel after 10 min and the 10% CP rinse after 1 min dislodged, by effervescence, much of the biofilm from the discs such that random sampling for CLSM was impossible.

**Table 1:** Area of biofilm remaining after application of the test agents

Test agents	Area (%) of biofilm remaining	SD
Control	89.6	9.8
1% Chlorhexidine gel		
After 1 min	95.5	3.8
After 3 min	91.9	3.1
After 10 min	93.9	0.9
		
5% Carbamide peroxide gel		
After 1 min	81.4	6.3
After 3 min	60.1	12.4
After 10 min	50.0	8.2
		
10% Carbamide peroxide gel		
After 1 min	60.2	4.1
After 3 min	45.0	22.4
After 10 min	0.7	0.2
5% Carbamide peroxide rinse 1 min	88.5	10.0
10% Carbamide peroxide rinse 1 min	1.7	2.5

### Bactericidal effects

**Fig. 3:**
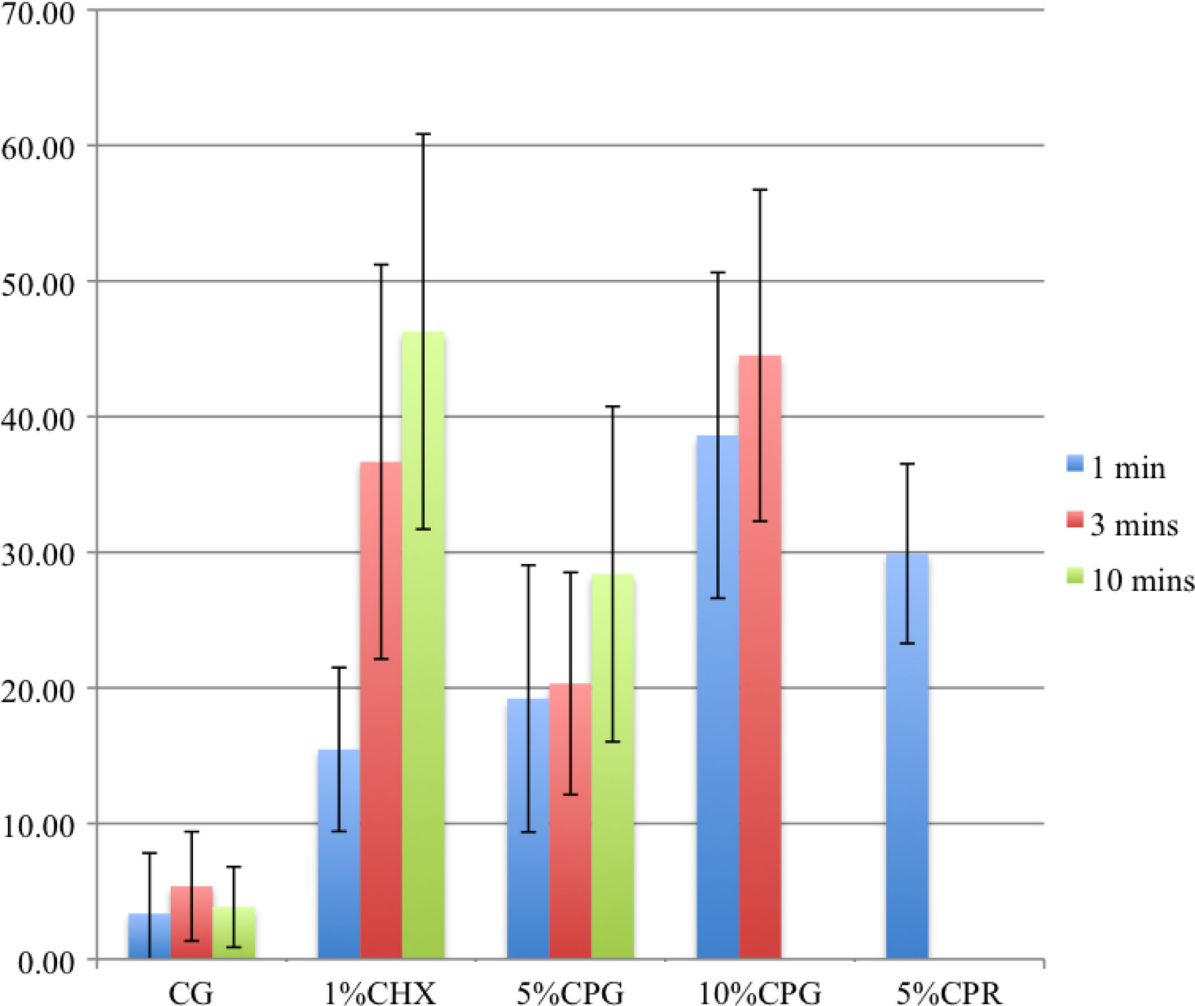
Bar graph representing percentage of dead bacteria after test agent application.

**Fig. 4:**
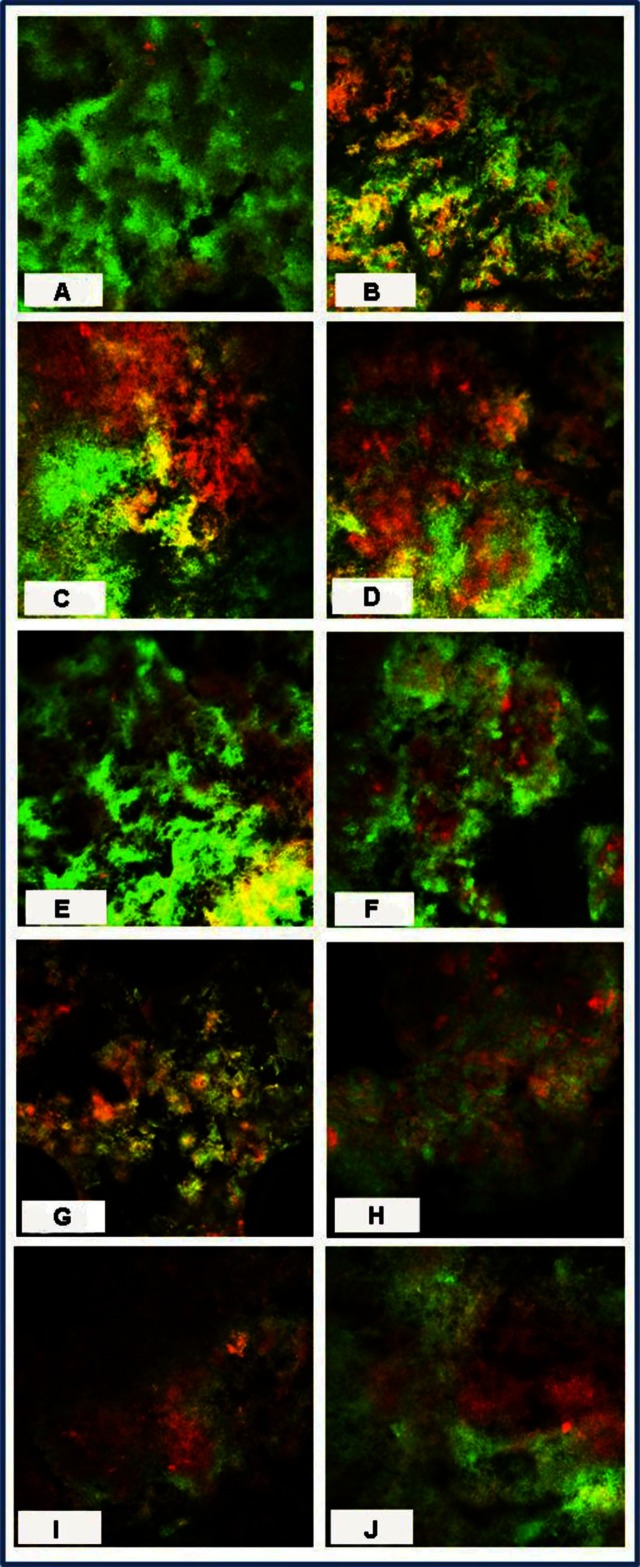
Confocal scanned segment of biofilm stained with LIVE/DEAD BacLight Bacterial Viability kit after exposure to (A) control, 1% CHX gel for (B) 1 min, (C) 3 mins, (D) 10 mins, 5% CP gel for (E) 1 min, (F) 3 mins, (G) 10 mins, 10% CP gel for (H) 1 min, (I) 3 mins, and (J) 5% CP rinse for 1 min.

All the CHX and CP agents reduced the amount of viable bacteria when compared to the CG (Table 2 and Figs. 3 and 4) and the reduction increased with the concentration and duration of the applications. CP at 10% was more bactericidal than at 5% for all immersion times. The 5% CP gel showed comparable bactericidal but slower acting effect to the CHX gel, whereas, the 5% CP rinse was faster acting but less bactericidal than the CHX gel. Overall, the 10% CP gel and rinse were both more bactericidal and faster acting than the CHX gel.

**Table 2:** The proportion of live bacteria to live-and-dead bacteria after applying the test agents

Test agents	% of live bacteria	SD
Control	94.8	4.2
1% Chlorhexidine gel 1 min	60.8	16.4
1% Chlorhexidine gel 3 min	56.3	15.0
1% Chlorhexidine gel 10 min	40.4	18.3
5% Carbamide peroxide gel 1 min	50.2	20.6
5% Carbamide peroxide gel 3 min	71.6	9.8
5% Carbamide peroxide gel 10 min	52.3	16.3
10% Carbamide peroxide gel 1 min	57.6	16.9
10% Carbamide peroxide gel 3 min	43.6	21.6
10% Carbamide peroxide gel 10 min	–	–
5% Carbamide peroxide rinse 1 min	49.0	13.8
10% Carbamide peroxide rinse 1 min	–	–

## Discussion

Anaerobes are the common cause of upper respiratory tract infections and are difficult to treat in older people. An antibacterial agent effective against oral anaerobes will help to reduce the health risks for people who are frail. The biofilm grown in our model under anaerobic conditions matures in about 3 weeks of incubation and after that does not change markedly (29). It has a heterogeneous mixture of cocci, rods, and filaments, with less viable cells at the basal layer, possibly due to nutrient deprivation (28). The proportion of live and dead cells varied throughout the biofilm thickness and could partly explain the high standard deviation values for the percentage of dead bacteria observed during the CLSM for all control and test groups.

Effervescence occurs when bacterial enzymes decompose CP and release oxygen and carbon dioxide. Decomposition kinetics of CP appeared to be dependent on the concentration, viscosity, and exposure time of the agent. Higher concentrations resulted in faster reaction and greater effervescence, causing more disruption to the biofilm. When the exposure time of 5% CP gel was increased to 10 min, about half of the disc surfaces had no visible biofilm. Effervescence from the 10% CP rinse dislodged the biofilm almost completely after 1 min, offering a clear advantage to the more passive CHX, which showed a comparable bactericidal effect. The gelation material increases the viscosity of the agents and allows prolonged contact with the oral tissues, which might inhibit the build-up of biofilm in the oral environment with saliva, gingival exudate, desquamation of cells, and food. Moreover, CP raises the pH of its environment as it decomposes, which could inhibit the growth of acidogenic bacteria, such as *S. mutans* (19). Therefore, we have confirmed our hypothesis that CP gels and rinses at 5% and 10% are comparable or superior than CG and 1% CHX gel in antibacterial and dislodging effects on oral biofilm.

There are limitations in this study. The HA disc mimics dental hard tissues chemically but not anatomically, which might influence the way the biofilm develops, proliferates, and is colonised. Nonetheless, it allows us to grow biofilm reproducibly (28). With CLSM, multiple scans of a single biofilm can be viewed quickly and under hydrated and undisrupted conditions (30). However, standardisation of the fluorescence is based on a balance between the intensity of red and green signals, which is established subjectively and varies with the thickness of the biofilm (31). The anaerobic species were not identified in our study, although previous studies established that biofilm cultured under anaerobic conditions consists of a mixture of bacteria (28). It is difficult to determine the bactericidal effect of different concentrations and exposures to HP since it varies with bacterial species, types of media used for bacterial culture, and treatment conditions (32).

People who are frail or institutionalised frequently have difficulties keeping their mouth and teeth clean and are at high risk of upper respiratory tract infections. CP could help them in reducing this risk. Additional studies are required to test the effects of CP and HP on human macrophages or on the specific pathogens associated with dental caries, periodontal disease, and aspiration pneumonia. Clinical studies using the split-mouth technique and the experimental gingivitis model, for example, could determine the anti-plaque effects of CP compared to a CG or CHX gel. Even more significantly, the CP gel could be compared to tooth brushing alone or to a CHX agent in reducing respiratory problems in institutionalised patients.

## Conclusion

CP gels and rinses at 5% and 10% concentrations, when compared to a control and a 1% CHX gel, had superior or comparable bactericidal and dislodging effects on oral biofilm cultured within an in vitro anaerobic model.
